# Number of Pairs of Natural Teeth, Rather Than Number of Present Teeth, Is Associated With Obesity Among Older Adults: A Cross‐Sectional Study

**DOI:** 10.1111/idh.70027

**Published:** 2025-12-07

**Authors:** Júlia Schmidt, Paulo Roberto Grafitti Colussi, Francisco Hecktheuer Silva, Diandra Genoveva Sachetti, Nathália Prigol Rosalen, Francisco Wilker Mustafa Gomes Muniz

**Affiliations:** ^1^ School of Dentistry, Universidade Federal de Pelotas Pelotas Rio Grande do Sul Brazil; ^2^ Graduation Program of Human Aging, University of Passo Fundo Passo Fundo Rio Grande do Sul Brazil; ^3^ Federal University of Pelotas Pelotas Rio Grande do Sul Brazil; ^4^ School of Dentistry. University of Passo Fundo Passo Fundo Rio Grande do Sul Brazil

**Keywords:** dental care for aged, epidemiology, nutrition disorders, tooth loss

## Abstract

**Objective:**

This study aimed to associate the number of present teeth and pairs of natural teeth with obesity in older adults (≥ 60 years old).

**Methods:**

This study used a probabilistic and representative sample from two southern Brazilian cities (Cruz Alta and Veranópolis). Data collection took place at the participants' households. Obesity was defined as body mass index ≥ 30Kg/m^2^. Oral examination was performed by calibrated researchers. Pairs of natural teeth were defined as pairs of antagonistic teeth. Sociodemographic, behavioural, medical, and dental history variables were also collected. Adjusted models were constructed using Poisson, considering different definitions of pairs of natural teeth (α < 5%). Independent analyses were performed for the whole sample and for each city.

**Results:**

The study included 559 individuals. Prevalence of obesity was 29.9%. Number of present teeth was 7.80 ± 8.68, while the total number of pairs of teeth was 2.16 ± 3.92. No significant association was detected between obesity and the number of teeth present (*p* = 0.254) or total pairs of natural teeth present (*p* = 0.065). However, for each pair of natural molars, the prevalence ratio (PR) of obesity was reduced by 22% (95% CI: 0.64–0.95). Similarly, for each functional tooth unit present, the PR for obesity was reduced by 4% (95% CI: 0.93–0.99). To the most significant associations, a weak effect size was detected. Similar results were detected in Veranópolis, but no significant association was observed in Cruz Alta.

**Conclusion:**

Higher numbers of pairs of natural molars, rather than the higher number of overall natural teeth, are associated with lower prevalence of obesity among older adults.

## Clinical Relevance

**Scientific Rationale for Study:**

Obesity is associated with lower number of present teeth. However, literature does not report the importance of groups of teeth in this association.

**Principal Findings:**

The number of present teeth may not be associated with obesity. However, the number of pairs of natural molars are associated with obesity.

**Practical Implications:**

It is suggested that the organization of teeth, especially the molar ones, has a greater effect on the occurrence of obesity rather than the number of teeth itself.

## 1 Introduction

The classification of obesity presents significant challenges; body compositions are constantly changing, and there are international differences in the onset age for this condition and rates of fat accumulation [1, 2]. The most prominent method for obesity classification is body mass index (BMI), which is widely used as an indicator of nutrition levels. Individuals with obesity are more prone to developing comorbid impairments such as diabetes mellitus, cancer, and coronary heart diseases, predisposing them to higher mortality risk [3]. Furthermore, it is crucial to study this condition, given that the literature indicated an annual increase in obesity of 0.48% from 2000 to 2019, with projections suggesting a further 39% increase from 2020 to 2030 [4].

Literature has also demonstrated an association between obesity and poor oral health, particularly in terms of dental caries [5] and periodontitis [6]. Significantly, dental caries and periodontitis are the most prevalent chronic oral diseases among adults [7, 8] and the main causes of tooth loss [9].

The presence of a higher number of teeth and a larger occlusal area results in better nutritional outcomes [10]. As the number of teeth present decreases, masticatory efficiency and performance can also decrease [11], with consequent impacts on the individual's body condition. In fact, the World Health Organization (WHO) defined a functional, aesthetic, and natural dentition at a minimum of 20 teeth [12]. However, the number itself does not consider the arrangement of the teeth and may not be the best measurement of chewing capacity [13]. In this context, the number of pairs of antagonistic teeth is also known to influence masticatory efficiency [14]. This topic also warrants investigation, as population‐based studies have revealed a high prevalence of tooth loss in Brazil [15].

Prior research has indicated the possibility of a bi‐directional association between tooth loss and obesity [16]. People with tooth loss tend to consume fewer vegetables and fruits and more foods with a high energy content. Furthermore, eating soft foods quickly reduces chewing capacity and, consequently, the chewing cycle, helping to increase BMI [17].

However, there remains a lack of research on the specific relationship between the number of pairs of natural teeth and body weight among older adults, necessitating further studies. Thus, the present study aimed to associate obesity with the total number of teeth and the number of pairs of natural teeth (and different categorization of pairs of occluding teeth) among older adults. The null hypothesis was that no statistically significant association between obesity and the number of pairs of natural teeth would be detected among older adults.

## 2 Material and Methods

### 2.1 Study Design and Location

This cross‐sectional study followed the STROBE checklist to interview and examine older adults, aged at least 60 years, from two southern cities of Brazil. The population of Veranópolis is approximately 22,810 inhabitants (289.397 km^2^), while the population of Cruz Alta is approximately 62,821 inhabitants (1360.548 km^2^) [18]. Both of them are located in the state of Rio Grande do Sul. Approximately 87% and 95% of the populations, respectively, live in the urban areas of these cities. The city of Veranópolis was firstly chosen, as it is recognised by the World Health Organization (WHO) as an “Age‐Friendly city” due to its longest life expectancy in Brazil (life expectancy at birth: 77.7) [19]. Meanwhile, Cruz Alta was chosen by convenience, as this city has a lower socioeconomical status when compared to Veranópolis, such as poorer Gini Index 0.5419 (in 2010) (Gini Index for Veranópolis was 0.4836 in 2010) and lower life expectancy at birth (76.49).

For each city, two distinct research protocols were developed, verified, and approved by the local ethics committee (University of Passo Fundo—protocol numbers CAAE 55099316.4.0000.5342 and 97924118.0.0000.5342). All participants read and signed an informed consent prior to their inclusion in the study.

### 2.2 Sample Size

The sample size calculation for the present study was based on the 25.3% prevalence of obesity (primary outcome) in older adults, as shown in the literature [20]. A 5% error (normal variable 1.96) and an absolute error of 5% were assumed. The total population of 3730 (aged 65 to 74 in Cruz Alta) was detected in the last available Brazilian census [18]. As the number of older adults in Veranópolis was lower (3493 aged at least 60 years [18]), this number was not considered in the current analysis.

Using these data, an alpha error of 5%, and a 95% confidence interval (95% CI), a total of 270 subjects per city was deemed necessary. Probabilistic sampling strategies were performed in each city to visit 300 households, regardless of the number of older adults living in each household.

### 2.3 Sample Strategy

To ensure that the sample was representative of both cities, different sampling strategies were employed for the cities of Veranópolis [21] and Cruz Alta [22]. Due to the high number of individuals in the urban area of Cruz Alta, only those living in that area were included. Cruz Alta has 68 districts, which were listed and numbered using maps provided by the city hall. From these, 17 (25%) were randomly drawn using the website www.random.org. The proportionality of residents for each area was respected.

In Veranópolis, by contrast, sampling methods included older adults living in both rural and urban areas. A total of 82 blocks (20% of the total number of blocks) in the urban area were randomly selected using the website www.random.org. A total of three rural communities were also randomly selected from 21 total rural communities. In the rural region, households were visited until at least 12 older adults had been interviewed and examined in each of the selected communities.

### 2.4 Eligibility Criteria

Individuals were considered eligible when they met the following inclusion criteria:

- Different age criterion was proposed. For Cruz Alta's sample, those aged 65–74 years (as proposed by the World Health Organization for surveys in oral health [23]). For the Veranóplis's sample, it included those with at least 60 years (it used the age criterion that defines older adults in developing countries);
- A resident in the selected household;
- Present physical, medical, and mental conditions that would allow for the required clinical and anthropometric examinations.

Other than the age criterion, similar methodological aspects were used in both cities. In each household, if more than one older adult met the inclusion criteria, all eligible residents were invited to participate in the study. If residents were absent on the day of data collection, two more contact attempts were made per household prior to exclusion. Long‐stay homes, commercial homes, uninhabited homes, and those merely visiting the selected households were excluded. Older adults who did not respond or whose caregivers reported the impossibility of participation were also excluded.

### 2.5 Clinical Examination and Interviews

Individuals were interviewed between July and August 2016 in Cruz Alta, and between December 2018 and January 2019 in Veranópolis. In both cities, the same questionnaire was applied; the questionnaire included sociodemographic, behavioural, medical, and dental history. The questionnaire was constructed using the questions of the PCATool‐SB Brazil [24].

Obesity was defined by BMI (weight [in Kg] divided by height [in m^2^]). Weight was verified using an electronic scale with a maximum weight of 200 kg and a sensitivity of 100 g. Height measurements were taken using a vertical anthropometric ruler, which was graduated in centimetres, fixed on the wall, and employed a mobile cursor. For each individual and each parameter, only one measurement was taken. These measurements were performed by the same trained and calibrated researcher. Training for anthropometric measurements was also performed at the University of Passo Fundo clinics of the School of Physical Education and Physiotherapy with older adults seeking treatment in the school. For the calibration process, two measurements were performed with a one‐week interval. Using a weighted kappa, weight (range ±100 g) and height (range ±0.01 mm) showed scores of 1.00 and 0.70, respectively.

Oral health variables were collected by counting the total number of natural teeth, the number of pairs of natural teeth in occlusion, and the use of dental prosthesis, according to the criteria of the WHO [23]. These examinations were performed with the aid of a wooden spatula under natural light. All present teeth were considered, with the exception of third molars. Teeth with the possibility of rehabilitation were counted as present. In each city, the examinations were performed by two teams of researchers; each team consisted of an oral examiner, an anthropometric examiner, and a note taker. All examiners were trained by the same study coordinator (PRGC) to ensure standardized data collection [10]. For teeth counting, the kappa coefficient for inter‐ and intra‐examiner reproducibility was > 0.8.

### 2.6 Outcome Definition

The dependent variable of the present study was obesity, which was defined as BMI ≥ 30 kg/m^2^. Individuals were categorised for analysis as either with obesity (BMI ≥ 30 kg/m^2^) or without obesity (BMI < 30 kg/m^2^). Due to the relatively small number of individuals with low weight (BMI < 18.5 kg/m^2^) (*n* = 10), these individuals were excluded from analysis.

### 2.7 Primary Exposure

The primary exposure variables in this study were the number of teeth present and the number of pairs of natural teeth present, as determined during the oral examination. The number of pairs of natural teeth was defined as pairs of natural antagonistic teeth in occlusion. A maximum of 14 pairs of natural teeth was considered. The following categories were created:

- *Pairs of natural anterior teeth*—the sum of the following pairs of teeth: 13–43, 12–42, 11–41, 21–31, 22–32, and 23–33.
- *Pairs of natural premolars*—the sum of the following pairs of teeth: 14–44, 15–45, 24–34, and 25–35.
- *Pairs of natural molars*—the sum of the following pairs of teeth: 17–47, 16–46, 26–36, and 27–37.
- *Pairs of natural posterior teeth*—pairs of natural premolars + pairs of natural molars.
- *Total pairs of natural teeth*—pairs of natural anterior teeth + pairs of natural premolars + pairs of natural molars.
- *Posterior chewing capacity*—(pairs of natural premolars × 1) + (pairs of natural molars × 2).
- *Functional Tooth Unit* (*FTU*)—(pairs of natural anterior teeth × 1) + (pairs of natural premolars × 1) + (pairs of natural molars × 2).

### 2.8 Independent Variables

Age was recorded in years, and sex was dichotomized into men and women. Ethnicity was classified as white or non‐white, with the latter including older adults who referred to themselves as black, brown, yellow, or Indigenous. Level of education was classified as low (illiterate participants with incomplete or complete elementary school), medium (incomplete or complete high school), or high (at least incomplete higher education). Marital status was classified as married or not married, with the latter composed of people who reported being single, divorced, or widowed. Participants were further grouped into retired or not retired.

Regarding general health conditions, self‐reported hypertension, diabetes, and cardiovascular disease were dichotomized into “yes” or “no” for each of these conditions. The use of medications divided the study participants into two groups: those who reported using one or more medications and those who did not report the use of any medications. Smoking exposure was classified into current smokers, former smokers, and non‐smokers. Alcohol exposure was classified as “yes” or “no,” with those who reported consuming alcohol frequently or in moderation considered alcohol users. Participants who used any type of dental prosthesis in at least one arch were included in the group using dental prosthesis.

### 2.9 Statistical Analysis

Independent analyses were performed for the whole sample and for each city. Only individuals who had sufficient information to record both BMI and pairs of natural teeth were included in this analysis. No missing data were identified. Bi‐ and multivariable analyses were performed using Poisson regression with robust variance in order to estimate the prevalence ratio (PR) and 95% CI for the association between obesity and the independent variables. Independent models were constructed for the number of present teeth and each pair of natural teeth. Co‐variates were used to fit the model.

Only the independent variables that presented a *p*‐value < 0.20 in the bivariable analysis were included as covariates in the multivariable model. However, the main exposures of the present study (number of present teeth and all categories of pairs of natural teeth) and the use of dental prosthesis were maintained in the final adjusted model regardless of the *p*‐value observed in the crude analysis. Moreover, as different age criteria were imposed in each selected city, age was also included in all adjusted models. In all analyses, multicollinearity tests were performed. All analyses were performed using SPSS software, version 21.0, for Windows, using an *α* < 0.05 and analysis of model modifications.

Literature has no clear indication for the cutoff points of effect size of PR. However, literature states that a relative risk of > 2 or < 0.5 and > 5 or < 0.2 may be considered strong or very strong, respectively [25]. This interpretation was adopted in the present study.

## 3 Results

Table 1 presents the characteristics of the 559 individuals aged at least 60 years who were ultimately included in the study. From these, 281 were from Veranópolis and 278 from Cruz Alta. The majority of participants were women (67.6%), self‐declared white (78.9%), retired (82.1%), married (55.8%), and reported a low level of education (72.8%). Hypertension was reported by 64.6% of participants, while 80.5% reported no diabetes and 85.9% reported no cardiovascular disease. Ranges of age in Cruz Alta were 65–74, while in Veranópolis it was 60–98. Regarding the main outcome of the present study, 29.9% were determined to have obesity. From these, 71 (25.3%) and 96 (34.5%) were from Cruz Alta and Veranópolis, respectively.

**TABLE 1:** Sociodemographic, behavioural, medical, and dental variables and their associations with obesity among older adults. Analyses were conducted for the whole sample and for each city.

Variable	*N* (%) or Mean ± SD	Whole‐sample			Cruz Alta			Veranópolis		
		Without obesity	With obesity	*p*	Without obesity	With obesity	*p*	Without obesity	With obesity	*p*
	Median (IQR)	(*n* = 392; 70.1%)	(*n* = 167; 29.9%)		(*n* = 210; 74.7%)	(*n* = 71; 25.3%)		(*n* = 182; 65.5%)	(*n* = 96; 34.5%)	
Age (in years)	Mean ± SD	70.64 ± 6.32	69.52 ± 5.66	0.058^b^	69.50 ± 3.55	68.68 ± 3.39	0.101^b^	71.95 ± 8.27	70.15 ± 6.82	0.138^b^
	Median (IQR) [minimum–maximum]	70.00 (66.00–74.00) [60–98]	68.00 (65.00–73.00) [60–89]		69.00 (66.00–73.25) [65–74]	68.00 (65.00–72.00) [65–74]		70.00 (66.00–77.00) [60–98]	69.50 (65.00–74.00) [60–89]	
Sex	Male	137 (34.9)	44 (26.3)	0.047^a^	76 (36.2)	25 (35.2)	0.882^a^	61 (33.5)	19 (19.8)	0.016^a^
	Female	255 (65.1)	123 (73.7)		134 (63.8)	46 (64.8)		121 (66.5)	77 (80.2)	
Skin colour	White	318 (81.1)	123 (73.7)	0.048^a^	153 (72.9)	39 (54.9)	0.005^a^	165 (90.7)	84 (87.5)	0.413^a^
	Non‐white	74 (18.9)	44 (26.3)		57 (27.1)	32 (45.1)		17 (9.3)	12 (12.5)	
Level of education	Low	282 (71.9)	125 (74.9)	0.355^a^	138 (65.7)	47 (66.2)	0.839^a^	144 (79.1)	78 (81.3)	0.450^a^
	Medium	58 (14.8)	27 (16.2)		37 (17.6)	14 (19.7)		21 (11.5)	13 (13.5)	
	High	52 (13.3)	15 (9.0)		35 (16.7)	10 (14.1)		17 (9.3)	5 (5.2)	
Marital Status	Married	218 (55.6)	94 (56.3)	0.883^a^	121 (57.6)	42 (59.2)	0.821^a^	97 (53.3)	52 (54.2)	0.890^a^
	Not married	174 (44.4)	73 (43.7)		89 (42.4)	29 (40.8)		85 (46.7)	44 (45.8)	
Retirement	Yes	321 (81.9)	138 (82.6)	0.833^a^	161 (76.7)	53 (74.6)	0.730^a^	160 (87.9)	85 (88.5)	0.877^a^
	No	71 (18.1)	29 (17.4)		49 (23.3)	18 (25.4)		22 (12.1)	11 (11.5)	
Hypertension	Yes	229 (58.4)	132 (79.0)	< 0.001^a^	123 (58.6)	57 (80.3)	< 0.001^a^	106 (58.2)	75 (78.1)	< 0.001^a^
	No	163 (41.6)	35 (21.0)		87 (41.4)	14 (19.7)		76 (41.8)	21 (21.9)	
Diabetes	Yes	63 (16.1)	46 (27.5)	0.002^a^	29 (13.8)	22 (31.0)	0.001^a^	34 (18.7)	24 (25.0)	0.218^a^
	No	329 (83.9)	121 (72.5)		181 (86.2)	49 (69.0)		148 (81.3)	72 (75.0)	
Cardiovascular disease	Yes	56 (14.3)	23 (13.8)	0.873^a^	21 (10.0)	8 (11.3)	0.761^a^	35 (19.2)	15 (15.6)	0.457^a^
	No	336 (85.7)	144 (86.2)		189 (90.0)	63 (88.7)		147 (80.8)	81 (84.4)	
Use of medicines	Yes	323 (82.4)	150 (89.8)	0.026^a^	169 (80.5)	63 (88.7)	0.113^a^	154 (84.6)	87 (90.6)	0.161^a^
	No	69 (17.6)	17 (10.2)		41 (19.5)	8 (11.3)		28 (15.4)	9 (9.4)	
Smoking exposure	Smokers	35 (8.9)	15 (9.0)	0.973^a^	19 (9.0)	13 (18.3)	0.092^a^	16 (8.8)	2 (2.1)	0.087^a^
	Former smokers	107 (27.3)	44 (26.3)		62 (29.5)	21 (29.6)		45 (24.7)	23 (24.0)	
	Never smokers	250 (63.8)	108 (64.7)		129 (61.4)	37 (52.1)		121 (66.5)	71 (74.0)	
Alcohol exposure	Yes	181 (46.2)	82 (49.1)	0.525^a^	82 (39.0)	26 (36.6)	0.716^a^	99 (54.4)	56 (58.3)	0.530^a^
	No	211 (53.8)	85 (50.9)		128 (61.0)	45 (63.4)		83 (45.6)	40 (41.7)	
Use of dental prothesis	Yes	338 (86.2)	139 (83.2)	0.360^a^	178 (84.8)	56 (78.9)	0.250^a^	160 (87.9)	83 (86.5)	0.728^a^
	No	54 (13.8)	28 (16.8)		32 (15.2)	15 (21.1)		22 (12.1)	13 (13.5)	

Abbreviations: IQR, interquartile range; SD, standard deviation.

^a^ Chi‐square.

^b^ Mann–Whitney.

Table 2 presents the mean numbers of present teeth and the different categories of pairs of natural teeth, divided between those with obesity and those without obesity. For the whole sample, this analysis determined that only the number of pairs of molars was significantly lower in individuals with obesity compared to those without obesity (*p* = 0.041). Similar results were detected in the city of Veranópolis, but no statistically significant difference for the number of natural teeth and pairs of teeth was observed in the city of Cruz Alta.

**TABLE 2:** Means (± standard deviation) of dental variable of non‐obese and obese individuals. Analyses were conducted for the whole sample and for each city.

Variable	Whole‐sample			Cruz Alta			Veranópolis		
	Without obesity (*n* = 392; 70.1%)	With obesity (*n* = 167; 29.9%)	*p*	Without obesity (*n* = 210; 74.7%)	With obesity (*n* = 71; 25.3%)	*p*	Without obesity (*n* = 182; 65.5%)	With obesity (*n* = 96; 34.5%)	*p*
Number of natural teeth	7.97 ± 8.87	7.69 ± 8.28	0.923^a^	8.31 ± 8.35	8.87 ± 7.89	0.452^a^	7.58 ± 9.45	6.81 ± 8.48	0.727^a^
Pairs of anterior natural teeth	1.34 ± 2.29	1.23 ± 2.14	0.952^a^	1.34 ± 2.25	1.32 ± 2.12	0.697^a^	1.33 ± 2.34	1.16 ± 2.16	0.745^a^
Pairs of premolar natural teeth	0.54 ± 1.15	0.50 ± 1.15	0.524^a^	0.53 ± 1.14	0.49 ± 1.13	0.678^a^	0.55 ± 1.17	0.51 ± 1.17	0.647^a^
Pairs of molar natural teeth	0.41 ± 1.00	0.23 ± 0.75	0.041^a^	0.32 ± 0.92	0.23 ± 0.70	0.742^a^	0.51 ± 1.08	0.23 ± 0.79	0.010^a^
Pairs of posterior natural teeth	0.95 ± 2.04	0.73 ± 1.74	0.333^a^	0.85 ± 1.94	0.72 ± 1.61	0.954^a^	1.06 ± 2.15	0.74 ± 1.84	0.180^a^
Total pairs of natural teeth	2.28 ± 4.08	1.96 ± 3.61	0.829^a^	2.19 ± 3.88	2.04 ± 3.39	0.617^a^	2.39 ± 4.31	1.90 ± 3.78	0.518^a^
Posterior chewing capacity	1.36 ± 3.00	0.96 ± 2.42	0.295^a^	1.17 ± 2.82	0.94 ± 2.20	0.932^a^	1.57 ± 3.19	0.97 ± 2.58	0.144^a^
Functional Tooth Unit	2.69 ± 4.96	2.19 ± 4.19	0.776^a^	2.51 ± 4.63	2.27 ± 3.89	0.610^a^	2.90 ± 5.30	2.13 ± 4.43	0.461^a^

^a^ Mann–Whitney (comparation between non‐obese and obese).

Table 3 presents the bivariable analysis results for the association between obesity and sociodemographic, medical, and dental history variables. None of the primary exposures were significantly associated with obesity in all analyses performed.

**TABLE 3:** Bivariable analysis for the association between obesity and sociodemographic variables, medical, and dental history among older adults. Analyses were conducted for the whole sample and for each city.

Variable	Whole‐Sample		Cruz Alta		Veranópolis	
	PR (95% CI)	*p*	PR (95% CI)	*p*	PR (95% CI)	*p*
Age	0.98 (0.96–1.00)	0.049	0.95 (0.90–1.01)	0.087	0.98 (0.96–1.00)	0.058
Sex						
Male	1	0.053	1	0.882	1	0.024
Female	1.34 (0.99–1.80)		1.03 (0.68–1.57)		1.64 (1.07–2.52)	
Ethnicity						
White	1	0.041	1	0.005	1	0.391
Non‐white	1.34 (1.01–1.77)		1.77 (1.19–2.63)		1.23 (0.77–1.96)	
Level of education						
Low	1		1		1	
Medium	1.03 (0.73–1.46)	0.848	1.08 (0.65–1.80)	0.766	1.09 (0.69–1.73)	0.720
High	0.73 (0.46–1.17)	0.187	0.88 (0.48–1.59)	0.662	0.65 (0.29–1.43)	0.280
Marital status						
Married	1	0.883	1	0.821	1	0.890
Not married	0.98 (0.76–1.27)		0.95 (0.63–1.44)		0.98 (0.71–1.35)	
Retirement						
Yes	1	0.834	1	0.728	1	0.878
No	0.83 (0.69–1.35)		1.09 (0.69–1.72)		0.96 (0.58–1.60)	
Diabetes						
Yes	1	0.001	1	< 0.001	1	0.202
No	0.64 (0.49–0.83)		0.49 (0.33–0.74)		0.79 (0.55–1.13)	
Cardiovascular disease						
Yes	1	0.874	1	0.758	1	0.469
No	1.03 (0.71–1.49)		0.91 (0.48–1.70)		1.18 (0.75–1.87)	
Use of medicines						
Yes	1	0.038	1	0.135	1	0.192
No	0.62 (0.40–0.97)		0.60 (0.31–1.17)		0.67 (0.37–1.22)	
Smoking exposure						
Smokers	1		1		1	
Former smokers	0.97 (0.59–1.59)	0.907	0.62 (0.36–1.09)	0.097	3.04 (0.79–11.72)	0.106
Never smokers	1.01 (0.64–1.58)	0.981	0.55 (0.33–0.91)	0.020	3.33 (0.89–12.45)	0.074
Alcohol exposure						
Yes	1	0.525	1	0.717	1	0.531
No	0.92 (0.72–1.19)		1.08 (0.71–1.64)		0.90 (0.65–1.25)	
Use of dental prothesis						
Yes	1	0.349	1	0.236	1	0.724
No	1.17 (0.84–1.63)		1.33 (0.83–2.15)		1.09 (0.68–1.73)	
Number of natural teeth	1.00 (0.98–1.01)	0.719	1.01 (0.98–1.03)	0.604	0.99 (0.98–1.01)	0.501
Pairs of anterior natural teeth	0.98 (0.93–1.04)	0.596	1.00 (0.91–1.09)	0.949	0.98 (0.91–1.05)	0.548
Pairs of premolar natural teeth	0.98 (0.87–1.10)	0.746	0.98 (0.81–1.18)	0.823	0.98 (0.85–1.13)	0.796
Pairs of molar natural teeth	0.83 (0.69–1.01)	0.060	0.90 (0.60–1.17)	0.425	0.78 (0.59–1.02)	0.070
Pairs of posterior natural teeth	0.96 (0.89–1.03)	0.245	0.97 (0.87–1.09)	0.598	0.95 (0.86–1.04)	0.250
Total pairs of natural teeth	0.99 (0.95–1.02)	0.370	0.99 (0.94–1.05)	0.762	0.98 (0.94–1.02)	0.353
Posterior chewing capacity	0.96 (0.91–1.01)	0.147	0.98 (0.90–1.06)	0.523	0.95 (0.88–1.02)	0.154
Functional tooth unit	0.98 (0.96–1.01)	0.247	0.99 (0.95–1.04)	0.675	0.98 (0.94–1.02)	0.236

Abbreviations: CI, confidence interval; PR, prevalence ratio.

Table 4 presents the adjusted analyses for the association between obesity, number of present teeth, and different categories of pairs of natural teeth. Only the primary exposures are shown. No statistically significant association was reported between obesity and the total number of present teeth (*p* = 0.254 [whole‐sample], *p* = 0.835 [Cruz Alta], and *p* = 0.124 [Veranópolis]). Conversely, in the whole sample, for each pair of natural molars, PR was 22% lower for the occurrence of obesity (95% CI: 0.64–0.95). Statistically significant associations were also detected between obesity and posterior chewing capacity (PR: 0.94; 95% CI: 0.88–0.99 [whole sample] and PR: 0.94; 95% CI: 0.90–0.99 [Veranópolis]) and FTU (PR: 0.96; 95% CI: 0.93–0.99 [whole sample] and PR: 0.89; 95% CI: 0.81–0.97 [Veranópolis]). These significant associations were considered weak. However, when only anterior or premolar pairs of natural teeth were considered, no statistically significant associations were detected (*p* > 0.05). Additionally, no statistically significant associations between the primary exposures and obesity were detected in Cruz Alta.

**TABLE 4:** Adjusted analysis of the association between obesity and the number of teeth present or functional tooth unit categories. Analyses were conducted for the whole sample and for each city.

Variable	Whole‐sample		Cruz Alta		Veranópolis	
	PR (95% CI)	*p*	PR (95% CI)	*p*	PR (95% CI)	*p*
Number of natural teeth	0.99 (0.97–1.01)^a^	0.254	1.00 (0.98–1.03)^b^	0.835	0.98 (0.96–1.01)^c^	0.124
Pairs of anterior natural teeth	0.96 (0.92–1.00)^a^	0.065	0.96 (0.86–1.06)^b^	0.409	0.94 (0.84–1.04)^c^	0.232
Pairs of premolar natural teeth	0.95 (0.82–1.09)^a^	0.436	0.98 (0.82–1.18)^b^	0.838	0.90 (0.73–1.10)^c^	0.303
Pairs of molar natural teeth	0.78 (0.64–0.95)^a^	0.013	0.92 (0.68–1.24)^b^	0.575	0.66 (0.49–0.88)^c^	0.006
Pairs of posterior natural teeth	0.92 (0.85–1.00)^a^	0.062	0.98 (0.86–1.10)^b^	0.681	0.86 (0.76–0.97)^c^	0.016
Total pairs of natural teeth	0.96 (0.92–1.00)^a^	0.065	0.98 (0.92–1.04)^b^	0.474	0.94 (0.89–0.99)^c^	0.046
Posterior chewing capacity	0.94 (0.88–0.99)^a^	0.030	0.98 (0.93–1.03)^b^	0.468	0.94 (0.90–0.99)^c^	0.015
Functional Tooth Unit	0.96 (0.93–0.99)^a^	0.032	0.98 (0.90–1.07)^b^	0.630	0.89 (0.81–0.97)^c^	0.008

Abbreviations: CI, confidence interval; PR, prevalence ratio.

^a^ Adjusted for: age, sex, diabetes, use of medicines, and use of dental prothesis.

^b^ Adjusted for: age, ethnicity, smoking exposure, diabetes, and use of dental prosthesis.

^c^ Adjusted for: age, sex, diabetes, smoking exposure, and use of dental prosthesis.

Table 5 presents a secondary analysis of the adjusted analyses for the association between obesity and different categorizations of pairs of natural teeth. For the whole sample, when considering the different cutoff points for total pairs of natural teeth, it was observed that older adults with ≥ 7 pairs of natural teeth had a PR 38% (95% CI: 0.39–0.98) lower than those with ≤ 6. Similar results were detected among those presenting ≥ 11 0.52 (PR: 0.52; 95% CI: 0.27–0.99 [whole sample] and PR: 0.44; 95% CI: 0.20–0.98 [Veranópolis]) pairs of natural teeth.

**TABLE 5:** Adjusted analyses for the association between obesity and different categorizations of pairs of natural teeth (PNT) among older adults. Estimates were provided for the comparison with a reference category using Poisson regression with robust variance. Analyses were conducted for the whole sample and for each city.

Variable	Comparison with controls	Whole‐sample		Cruz Alta		Veranópolis	
		PR (95% CI)	*p*	PR (95% CI)	*p*	PR (95% CI)	*p*
Total PNT	≥ 1	0.93 (0.67–1.29)^a^	0.668	1.08 (0.70–1.68)^b^	0.728	0.83 (0.51–1.36)^c^	0.454
Total PNT	≥ 2	0.89 (0.63–1.26)^a^	0.523	0.96 (0.61–1.52)^b^	0.871	0.83 (0.50–1.37)^c^	0.463
Total PNT	≥ 3	0.82 (0.57–1.18)^a^	0.275	0.90 (0.55–1.45)^b^	0.656	0.74 (0.43–1.26)^c^	0.267
Total PNT	≥ 4	0.74 (0.50–1.11)^a^	0.147	0.85 (0.51–1.43)^b^	0.544	0.61 (0.34–1.09)^c^	0.096
Total PNT	≥ 5	0.77 (0.51–1.17)^a^	0.222	0.87 (0.51–1.48)^b^	0.600	0.68 (0.38–1.21)^c^	0.187
Total PNT	≥ 6	0.71 (0.46–1.09)^a^	0.118	0.79 (0.44–1.41)^b^	0.426	0.63 (0.34–1.14)^c^	0.125
Total PNT	≥ 7	0.62 (0.39–0.98)^a^	0.040	0.75 (0.38–1.49)^b^	0.415	0.51 (0.27–0.93)^c^	0.028
Total PNT	≥ 8	0.62 (0.38–1.02)^a^	0.059	0.69 (0.32–1.52)^b^	0.360	0.52 (0.27–1.01)^c^	0.053
Total PNT	≥ 9	0.60 (0.35–1.02)^a^	0.060	0.62 (0.23–1.69)^b^	0.348	0.50 (0.25–0.97)^c^	0.040
Total PNT	≥ 1 0	0.54 (0.29–0.99)^a^	0.046	0.52 (0.17–1.60)^b^	0.255	0.50 (0.24–1.04)^c^	0.062
Total PNT	≥ 11	0.52 (0.27–0.99)^a^	0.049	0.65 (0.22–1.90)^b^	0.431	0.44 (0.20–0.98)^c^	0.046
Total PNT	≥ 12	0.64 (0.31–1.30)^a^	0.215	0.95 (0.34–2.63)^b^	0.916	0.51 (0.20–1.32)^c^	0.166
Total PNT	≥ 13	0.43 (0.17–1.12)^a^	0.082	0.41 (0.06–2.72)^b^	0.357	0.49 (0.16–1.50)^c^	0.215
Total PNT	14	0.91 (0.37–2.21)^a^	0.831	0.86 (0.15–4.88)	0.863	0.89 (0.30–2.66)^c^	0.836
Molar PNT	≥ 1	0.58 (0.37–0.91)^a^	0.018	0.95 (0.50–1.82)	0.877	0.31 (0.17–0.57)^c^	< 0.001
Molar PNT	≥ 2	0.50 (0.28–0.91)^a^	0.023	0.72 (0.27–1.92)	0.516	0.38 (0.18–0.80)^c^	0.012
Molar PNT	≥ 3	0.38 (0.16–0.89)^a^	0.026	0.67 (0.17–2.68)	0.573	0.33 (0.11–0.94)	0.038
Molar PNT	4	0.57 (0.23–1.44)^a^	0.235	0.57 (0.09–3.50)	0.546	0.62 (0.21–1.82)	0.379
Posterior PNT	≥ 1	0.80 (0.56–1.14)^a^	0.218	1.04 (0.65–1.66)	0.882	0.55 (0.30–0.99)	0.045
Posterior PNT	≥ 2	0.78 (0.51–1.17)^a^	0.226	1.09 (0.62–1.93)	0.764	0.52 (0.28–0.94)	0.029
Posterior PNT	≥ 3	0.73 (0.46–1.13)^a^	0.158	0.96 (0.49–1.85)	0.896	0.54 (0.29–0.99)	0.046
Posterior PNT	≥ 4	0.61 (0.35–1.05)^a^	0.072	0.72 (0.32–1.61)	0.423	0.47 (0.24–0.92)	0.028
Posterior PNT	≥ 5	0.60 (0.33–1.09)^a^	0.091	0.55 (0.19–1.63)	0.278	0.63 (0.31–1.30)	0.212
Posterior PNT	≥ 6	0.58 (0.28–1.17)^a^	0.128	0.85 (0.31–2.37)	0.759	0.47 (0.18–1.21)	0.118
Posterior PNT	≥ 7	0.40 (0.16–1.03)^a^	0.058	0.39 (0.060–2.57)	0.329	0.45 (0.15–1.37)	0.158
Posterior PNT	8	0.86 (0.36–2.09)^a^	0.742	0.78 (0.14–4.42)	0.780	0.89 (0.30–2.66)	0.836

Abbreviations: CI, confidence interval; PR, prevalence ratio.

^a^ Adjusted for: age, sex, diabetes, use of medicines, and use of dental prothesis.

^b^ Adjusted for: age, ethnicity, smoking exposure, diabetes, and use of dental prosthesis.

^c^ Adjusted for: age, sex, diabetes, smoking exposure, and use of dental prosthesis.

When considering the different cutoff points for pairs of natural molars, in the whole sample, a PR decrease of 42% (95% CI: 0.37–0.91) was observed in participants with at least one pair of molars compared to participants without any pair of molars. Similar results were detected in the city of Veranópolis and the whole sample analysis. Conversely, Cruz Alta did not show a significant association in any analyses. Regarding the size effect, strong magnitudes of the associations were detected for total pairs of natural teeth ≥ 5 (in Veranópolis), pairs of natural molars ≥ 1 (in Veranópolis), ≥ 2 (in Veranópolis), and ≥ 3 (in both whole sample and Veranópolis). Additionally, a strong association was observed for posterior pairs of natural teeth ≥ 4. All other significant associations were considered weak.

## 4 Discussion

This study aimed to evaluate the association between pairs of natural teeth and obesity among older adults. The assessment of this association can aid in the treatment or prevention of obesity‐related diseases and tooth loss. The study results demonstrated that the number of present teeth and total pairs of teeth were not significantly associated with obesity. However, when higher numbers of pairs of natural molars were detected, a significantly lower occurrence of obesity was also observed. Similar results were observed for FTU. These results suggest that the organization of teeth may have a greater effect on obesity than the sheer number of present teeth.

These results were very similar for the whole‐sample analyses and for Veranópolis, while, in Cruz Alta, no significant association was observed. Readers must consider the higher prevalence of obesity and complete edentulism in Veranópolis (34.5% and 48.6%, respectively) when compared to Cruz Alta (25.3% and 30.0%, respectively) when interpreting these results. Significantly higher mean age of the participants in Veranópolis (71.42 ± 7.90; interquartile range [IQR]: 65–76), when compared to Cruz Alta (69.30 ± 3.52; interquartile range [IQR]: 66–73) (*p* < 0.032), may also justify these findings. Literature shows that tooth loss is marked by aging, thus, influencing the current results [26].

Prior research has demonstrated that obesity tends to decrease with advancing age [27, 28]. During the aging process, significant changes occur in muscle mass and pattern of fat distribution, and these factors may explain the lower prevalence of obesity among older adults [29]. In addition, this decrease in obesity may be justified by the senescence process and the effects of survival bias, as obesity‐related diseases contribute to higher mortality [30]. However, it is important to highlight that a high prevalence of obesity was detected within the sampled population, and that the specific Brazilian demographic context must be considered when analyzing these results.

Regarding the definition of functional dentition, there is a lack of consensus, and different measurements can be found in the literature. The WHO criterion for functional dentition prevailed among studies until 2010 when aspects of occlusion and aesthetics began to be implemented in the assessment of dental configurations [31]. For example, the Eichner classification considers natural and restored teeth in bilateral contacts [32], while the FTU system attributes different values to natural tooth groups [33]. There is evidence to include periodontal status as well [34], supporting the importance of the presence of functional teeth. This variety of definitions can lead to misunderstandings about the real oral health status and may even confuse the effects of tooth loss. Nonetheless, it is clear that the assessment of masticatory capacity should consider pairs of teeth and their function in the dental arch instead of viewing teeth as isolated units.

Literature has reported a positive relationship between higher numbers of FTU and greater numbers of pairs of antagonistic teeth with better masticatory efficiency [35]. The masticatory function is the first oral stage of the digestion process, characterized by the sum of masticatory cycles. The lack of teeth implies a change in the ability to cut, crush, and tear food to allow for effective digestion [36]. It may be expected that individuals with lower numbers of pairs of natural teeth perform a higher number of chewing cycles; however, research has demonstrated that such individuals actually perform the same number of chewing cycles as those who retain all pairs of natural teeth [36, 37]. Therefore, individuals with fewer pairs of antagonistic teeth may swallow their food in comparatively larger particles.

In addition, individuals with obesity tend to have high consumption of fermentable carbohydrates coupled with reduced salivary flow, both of which are causal factors for dental caries [38]. Adipose tissues are present in large quantities in individuals with obesity; these tissues secrete several immunomodulating factors, including tumour necrosis factor‐alpha and adipocytokines, which can affect the periodontal response or contribute to periodontal diseases [39]. Dental caries and periodontitis are the primary oral diseases that lead to tooth loss and, consequently, to a lower number of pairs of natural teeth [9]. However, it is important to emphasise that these oral diseases were not assessed in the present study, representing a limitation of the study.

In this study, only pairs of natural teeth (particularly molar pairs) were significantly associated with lower rates of obesity. This group of teeth has a larger surface (or area of occlusal contact), with the first and second molars being responsible for providing 36.7% and 28%, respectively, of the entire effective chewing area. In comparison, the first and second premolars correspond to only 8% each [37]. Thus, these findings corroborate the conclusion that the most important factor in controlling masticatory performance is the amount of occlusal contact, while anterior teeth seem to play a secondary role in this scenario. However, it must be noted that third molars were not counted in the present study, presenting another limitation.

Masticatory efficiency is reduced in individuals with fewer teeth and in those with dental prostheses replacing natural teeth [40]. In the adjusted analysis of the present study, the use of dental prosthesis was not significantly associated with obesity (data not shown). It is important to emphasize that this study did not evaluate the quality of dental prostheses observed. However, prior research has suggested that artificial or even devitalized teeth cannot be expected to present the same function when compared with natural teeth [32]. Due to instability, lack of retention, and poor adaptation, dentures were considered poor substitutes for natural teeth in chewing food [14].

Literature has also reported that older adults progressively reduce their intake of hard‐to‐chew foods such as vegetables, fruits, and meat, replacing them with an easy‐to‐chew diet low in minerals and vitamins. This tendency may contribute to muscle atrophy [41]. Furthermore, research has demonstrated that individuals who wear dentures have a lower ability to grind food compared to individuals with natural teeth [36, 42]. On the other hand, individuals with dentures that are new, are in good condition, or have undergone adjustment show improvement in masticatory efficiency when compared to individuals with deficient dentition [43].

This study involved a representative sampling of two cities using a probabilistic sampling strategy. In addition, researchers were trained and calibrated to ensure the reliability of the data collected. However, the study's cross‐sectional design does present certain limitations, among them the inability to assess either the temporality of the associations between obesity and the independent variables or the quality of the observed dental prostheses. In addition, the timeframe of 2 years for data collection between the two cities must be considered when interpreting the results of the present study. Differences in the prevalence of both outcomes and exposures may not be ruled out, which may have inferred in the current results. Because different research protocols were developed for each city, different age ranges were involved between the two, which should also be understood as a limitation. Finally, the findings of this study should be limited to older adults, as other age groups are expected to have different occurrences of obesity and tooth loss, which would imply different estimates.

## 5 Conclusion

This study concluded that a higher number of pairs of natural molars is associated with a lower prevalence of obesity among older adults. By contrast, these findings were not detected for the overall number of natural teeth present.

## Author Contributions

F.W.M.G.M. and P.R.G.C. designed the study. P.R.G.C., D.G.S., and N.P.R. collected the data. F.W.M.G.M. analysed the data. J.S. and F.H.S. validated the results. J.S. and F.H.S. wrote the manuscript. All authors revised the literature, corrected the manuscript and approved its final version.

## Funding

This study was financed in part by the Coordinação de Aperfeiçoamento de Pessoal de Nível Superior—Brasil (CAPES)—Finance Code 001. All other funding was self‐supported by the authors.

## Conflicts of Interest

The authors declare no conflicts of interest.

## Data Availability

Data will be available upon request from the authors.
